# Research on the correlation between artificial intelligence and depression: A bibliometric analysis from 2000 to 2024

**DOI:** 10.1097/MD.0000000000050781

**Published:** 2026-09-18

**Authors:** Chunrong He, Junkang Zhao

**Affiliations:** a Mental Health Education and Counseling Center, Taiyuan Institute of Technology, Taiyuan, Shanxi, China; b Third Hospital of Shanxi Medical University, Shanxi Bethune Hospital, Shanxi Academy of Medical Sciences, Tongji Shanxi Hospital, Taiyuan, Shanxi, China.

**Keywords:** artificial intelligence, bibliometrics, citespace, depression, visualization

## Abstract

This study aims to systematically assess the research productivity of artificial intelligence (AI) technologies and depression using bibliometric methods, while also exploring the trends and prospects of AI applications in the diagnosis, prediction, and treatment of depression. This study employs bibliometric analysis, using the Web of Science database to collect relevant literature on depression and AI from 2000 to 2024. By analyzing indicators such as changes in the number of publications, keyword co-occurrence, author collaboration networks, and academic impact evaluation, the study comprehensively assesses the research dynamics and key topics in this field. The literature data are analyzed using visualization tools to identify core research themes and future development trends. From 2000 to 2024, the number of studies combining depression and AI showed steady growth. This study identified a total of 1437 papers published in 766 academic journals. Keyword analysis revealed that machine learning, deep learning, natural language processing, chatbots, and neuromorphic computing are the primary technological approaches used in this research. Regarding application domains, publications were categorized into screening (173 articles), diagnosis (279 articles), and treatment (944 articles), with a smaller group covering other aspects (41 articles). Additionally, international collaborative research has increased year by year, with particularly prominent scientific activities observed in the United States, China, and the United Kingdom. AI technology has demonstrated significant potential in the research and application of depression, particularly in precise diagnosis, personalized treatment, and early intervention. Nevertheless, key research gaps persist; long-term efficacy evidence is scarce, lack of standardized validation, and cross-disciplinary integration remains insufficient. This study employs bibliometrics analysis to map the field’s evolution, identifying these critical areas requiring focused research and collaboration to fully harness AI’s potential against depression.

## 1. Introduction

Depression is a pervasive mental health issue that profoundly impacts the quality of life for millions of individuals worldwide.^[1,2]^ It not only affects patients’ emotional and cognitive functioning but also significantly impairs their social functioning and life satisfaction.^[3]^ According to the World Health Organization, depression has emerged as one of the leading causes of disability globally.^[4]^ This situation necessitates more effective methods for diagnosing and treating depression to enhance patients’ quality of life and social adjustment.

The rapid advancement of artificial intelligence (AI) technology offers new opportunities for researching and treating depression.^[5]^ AI has demonstrated remarkable capabilities in data processing and pattern recognition, particularly within the healthcare field, and holds broad prospects for application.^[6]^ AI technologies, such as machine learning (ML) and natural language processing, have been utilized to analyze depressive symptoms, predict disease progression, and evaluate treatment efficacy.^[7,8]^ For instance, by analyzing extensive clinical data, patient speech patterns, and content from social media platforms, AI can provide personalized diagnostic recommendations and treatment options. Furthermore, AI can assist doctors in conducting more accurate risk assessments and early warnings, thereby improving depression management and intervention.^[9,10]^ However, realizing the full potential of AI for depression necessitates a specific focus on the disease itself. Depression’s inherent complexity, including its heterogeneity in mechanisms, presentations, and treatment responses, demands that AI applications be tailored to capture disease-specific nuances. A disease-centric perspective ensures research directly addresses the unique challenges faced by individuals with depression, guiding efforts towards clinically relevant outcomes. While AI research in depression is rapidly expanding, a significant gap persists: the lack of clear, unified definitions for AI’s specific applications and boundaries. Disagreement persists on AI’s role (e.g., diagnostic aid, risk prediction, therapy support) and how to measure its effectiveness. This ambiguity complicates comparative studies, standardized evaluation, and effective tool development, leading to clinical confusion and resource misallocation. Therefore, systematically mapping the existing literature to identify current research trends and pinpoint these definitional ambiguities is crucial.

Bibliometrics is a discipline that studies academic literature and its impact, dissemination, and usage patterns.^[11]^ It utilizes statistical and mathematical methods to quantitatively analyze the characteristics of literature, thereby revealing the dynamics and trends of scholarly communication.^[12–14]^ The main topics in bibliometrics include the analysis of literature production, citation analysis, author and institutional output analysis, journal impact evaluation, and the study of the distribution of keywords and research themes.^[15]^ These analytical tools and methods are widely applied in areas such as research evaluation, information management, and academic trend forecasting.^[16]^ Through a quantitative perspective, bibliometrics helps researchers and administrators understand the complexity of academic activities, optimize resource allocation and decision-making, and support the sustainable development of scientific research.^[17,18]^ Accordingly, conducting a bibliometric analysis at the intersection of AI and depression is crucial.

Our paper employs this approach to systematically analyze and synthesize the existing research landscape indexed in the Web of Science Core Collection (WoSCC). By utilizing visual analysis software and adopting a mixed-methods approach that integrates both qualitative and quantitative analysis, we can delve deeply into the correlation between depression and AI. This analysis provides practical value by identifying key knowledge clusters, emerging trends, and research gaps. This information can guide researchers towards underexplored areas or highlight promising methodological approaches, assist clinicians in preparing for future AI applications, and inform policymakers regarding resource allocation, public health strategies, and regulatory considerations for AI in depression. Ultimately, this study aims to facilitate the translation of AI advancements into tangible benefits for depression care and patient outcomes.

## 2. Method

### 2.1. Data collection

The WoSCC by Thomson Reuters indexes over 12,000 influential academic journals, which are widely regarded as important by the international academic community.^[19]^ The study retrieved literature indexed in WoSCC published between 2000 and 2024. The complete search query used was as follows: (TS = (“Depression”) OR TS = (“Depressive Symptoms”) OR TS = (“Depressive Symptom”) OR TS = (“Symptom, Depressive”) OR TS = (“Emotional Depression”) OR TS = (“Depression, Emotional”)) AND (TS = (“AI”) OR TS = (“artificial intelligence”)), language = English. The retrieved bibliographic records were downloaded and saved as plain text files in the format of “Full Record and Cited References” to serve as the sample for the analysis in this study. A total of 1437 bibliographic records were collected, named data. In addition, raw data regarding the countries/regions, institutions, journals, authors, and article types of the publications were gathered, and statistical analysis was performed using Microsoft Excel. Since this study only utilized publicly available data from published literature and did not involve human subjects, ethical approval and informed consent were not required.

### 2.2. Bibliometric analysis tools

#### 2.2.1. CiteSpace

CiteSpace software was utilized to construct a co-occurrence network, enabling the visualization of collaborative relationships and key scientific concepts within the research field. In this network, nodes represent entities such as institutions, countries, authors, or publications, depending on the dataset configuration. The size of each node reflects its occurrence frequency: for institutions, countries, or authors, this indicates the number of publications; for publications, it indicates citations received. Nodes are color-coded chronologically (white to red for years 2000–2024), and edges signify collaborations or co-occurrences. The thickness of concentric “tree rings” within nodes shows yearly occurrence frequency. The total number of nodes reported in the results provides a quantitative measure of the entities identified in the network analysis.

#### 2.2.2. HistCite

Histcite is literature indexing and analysis software used to process literature indexing information output from Web Of Science. It helps us to quickly grasp the historical development of the literature in a particular field and to identify key studies and key scholars. It also makes it easy to map out the historical relationships of the literature in the field, so that the development of the field, its relationships, and the people in it can be seen at a glance. The software utilizes 2 primary citation metrics: the local citation score and the global citation score. The local citation score represents the number of times a publication is cited within the dataset being analyzed, indicating its influence within that specific context. The global citation score reflects the total citation count of a publication across the entire WoSCC database, signifying its broader impact.

#### 2.2.3. The alluvial generator

To visualize the temporal evolution of research themes, we first employed CiteSpace to generate keyword co-occurrence networks segmented by time slices, capturing relationships among keywords within each period. Subsequently, these networks were imported into Alluvial Generator. This tool enables the clustering of keywords with commonalities into distinct “modules,” representing specific research foci during particular time periods. Through the generated alluvial diagram, it is possible to visually trace the evolutionary trajectories of these modules over time: they may remain stable, differentiate, merge into new modules, or gradually diminish in prominence. The width of each module in the diagram typically reflects its relative importance during that period. This visualization method clearly reveals the dynamic shifts in research themes, their structural dynamics, and their temporal associations and transformations, providing a foundation for subsequent analysis of the composition and evolution of specific modules.

#### 2.2.4. VOSviewer

VOSviewer is renowned for its probability-based data standardization method, which aids in more accurately revealing the strength of associations within the data. The software offers diverse visualization views, enabling intuitive presentation of various network relationships, such as keywords, co-institutions, and coauthors. Specifically, its visualization capabilities include network views, overlay views, and density views.

## 3. Results

### 3.1. The historical features of the literature on AI in depression

#### 3.1.1. Distribution of publications

The variation in the number of scientific publications at specific time points can reveal the accumulation of knowledge in a particular research field, providing important parameters for understanding the development of that field from a quantitative perspective.^[20]^ A total of 1437 publications related to AI and depression were retrieved for this study, including 1243 research articles and 194 review articles. The study involved 7465 authors and 2513 institutions, and the publications were distributed across 766 journals in 180 scientific categories.

**Figure 1.**
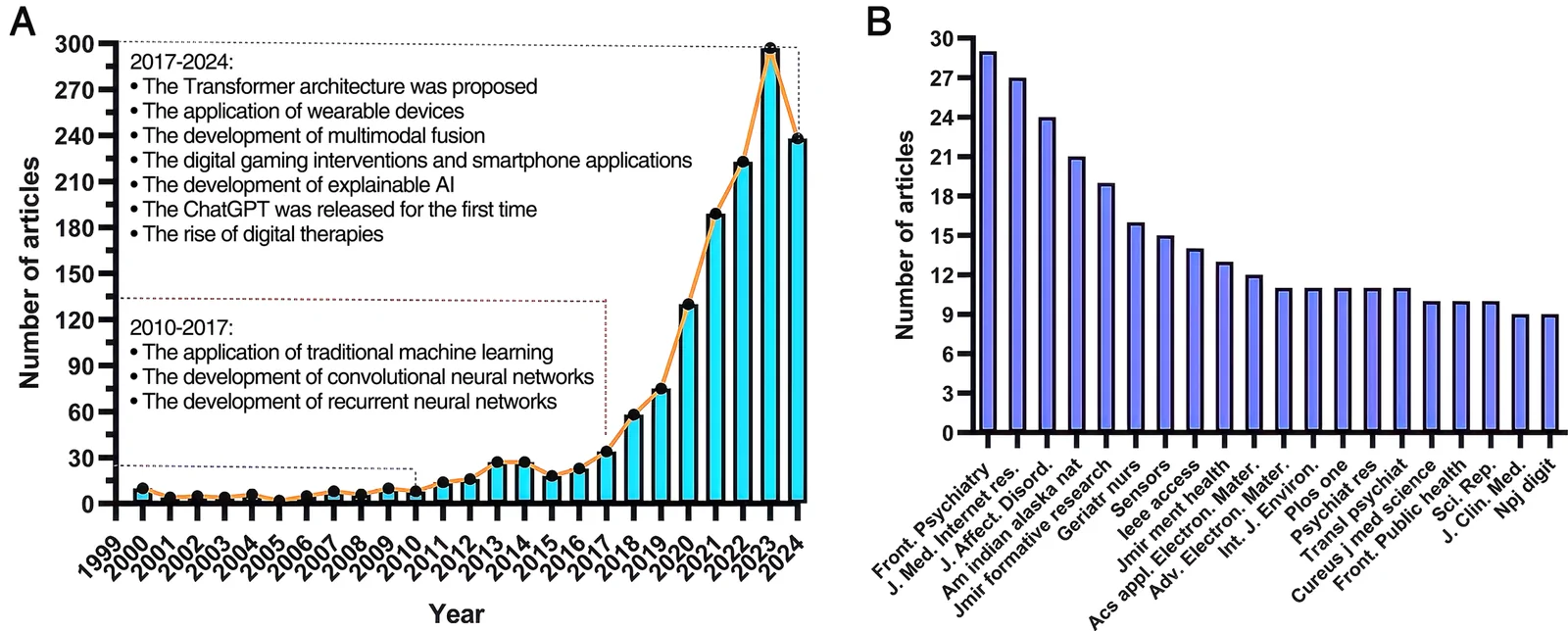
(A) The annual number of publications on AI and depression and the evolution of AI tools (2000–2024). (B) The top 20 journals with the highest publication volume on AI and depression. AI = artificial intelligence.

The annual publication trends, alongside the evolution of AI tools, are illustrated in Figure 1A. The number of publications remained relatively low from the start of the observation period until 2010, indicating a nascent stage for this research area. Between 2011 and 2017, the number of publications began to gradually increase. This growth phase coincided with significant developments in AI tools. Specifically, during this period, the application of traditional ML became more widespread, alongside the development of convolutional neural networks and recurrent neural networks, which began to offer more sophisticated data processing capabilities. The period from 2017 onwards witnessed a marked acceleration in the publication volume, culminating in a rapid surge after 2019 and peaking in 2023. This dramatic increase strongly correlates with a wave of transformative AI advancements. Key developments during this later phase included the proposal and widespread adoption of the Transformer architecture, which revolutionized natural language processing and beyond. Furthermore, the application of wearable devices for continuous health monitoring, the development of multimodal fusion techniques to integrate diverse data types, the emergence of digital gaming interventions and smartphone applications for mental health support, and the development of explainable AI to address the “black box” problem all gained significant traction. A pivotal moment was the release of ChatGPT in late 2022, which dramatically increased public and professional awareness of AI’s potential. Collectively, these advancements, along with the broader rise of digital therapies, likely contributed to the intensified research focus and the subsequent surge in publications observed after 2018.

The publication volume of Frontiers in Psychiatry ranks first with 29 papers, followed by Journal of Medical Internet Research with 27 papers and Journal of Affective Disorders with 24 papers. Figure 1B presents a list of the top 20 journals with the highest number of publications.

#### 3.1.2. Distribution of research domains

To comprehensively understand the landscape of AI applications in depression research, we analyzed the distribution of studies across key application domains based on titles, abstracts, and keywords. Regarding the distribution across key application domains, publications were categorized into 4 main groups: screening, diagnosis, treatment, and other aspects. The analysis yielded 173 articles on screening, 279 on diagnosis, and 944 on treatment. A smaller group of 41 articles covered other aspects. The relatively small number of publications related to rehabilitation limited the scope of analysis for this domain, and these were consequently included in an “other aspects” category for broader comparison where applicable. This categorization reveals a strong emphasis on AI applications in the treatment phases of depression.

#### 3.1.3. The vein of research on AI and depression

**Figure 2.**
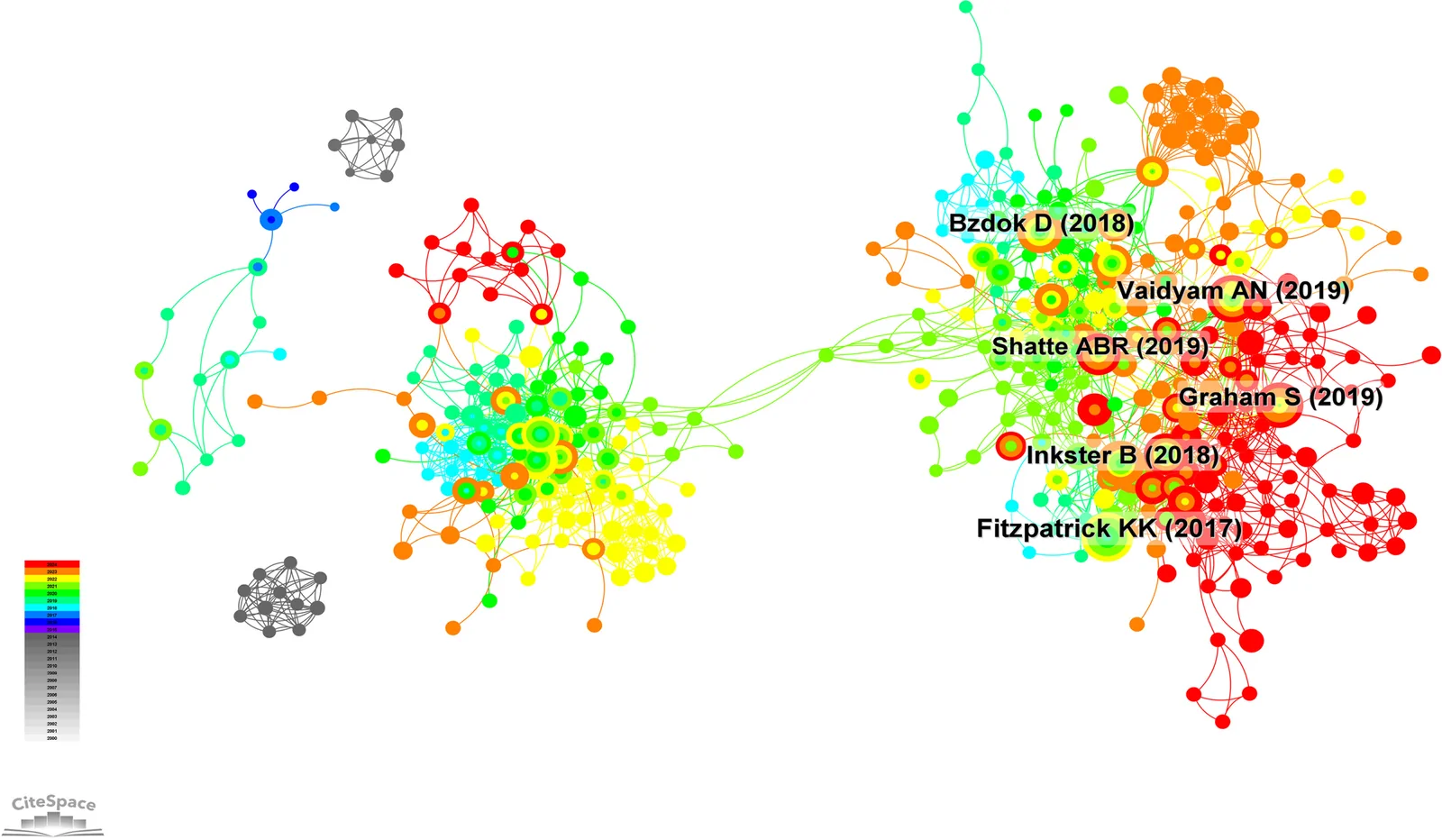
Co-citation chart of literature on AI and depression. AI = artificial intelligence.

The citation network graph illustrates the relationship between AI and depression over the past 2 decades, as depicted in Figure 2. The network consists of 895 nodes and 2776 links, indicating extensive connections within the literature in this research area. The nodes marked in gray in the early literature (2000–2010) formed the foundation of the field due to their node density and inter-node connections, providing essential support for its sustainable development. In the mid-term literature (2011–2017), the nodes marked in blue gradually dispersed, forming the primary branches of the research. In the later literature (2018–2024), the nodes developed into branches and formed tighter clusters, indicating increasing concentration and differentiation within the research field. Among these papers, those by Fitzpatrick KK (2017), Vaidyam AN (2019), Bzdok D (2018), Inkster B (2018), Graham S (2019), Shatte ABR (2019), Fulmer R (2018), Abd-alrazaq AA (2019), and van de Burgt Y (2017) are notably prominent, with total citation frequencies of 35, 31, 30, 30, 29, 28, 26, 23, and 22, respectively. These 9 papers occupy an important position in the field of AI and depression research. The concentration and differentiation within this research cluster will become clearer in the subsequent reference timeline.

Additionally, we used HisCite Pro 2.1 to construct the citation history map of the research articles. Table S1, Supplemental Digital Content 1 highlights these landmark articles (see Table S1, Supplemental Digital Content 1 online). By applying both methods, we were able to visually observe the citation network structure of the literature and identify the most influential articles in the field.

#### 3.1.4. Scientific cooperation

**Figure 3.**
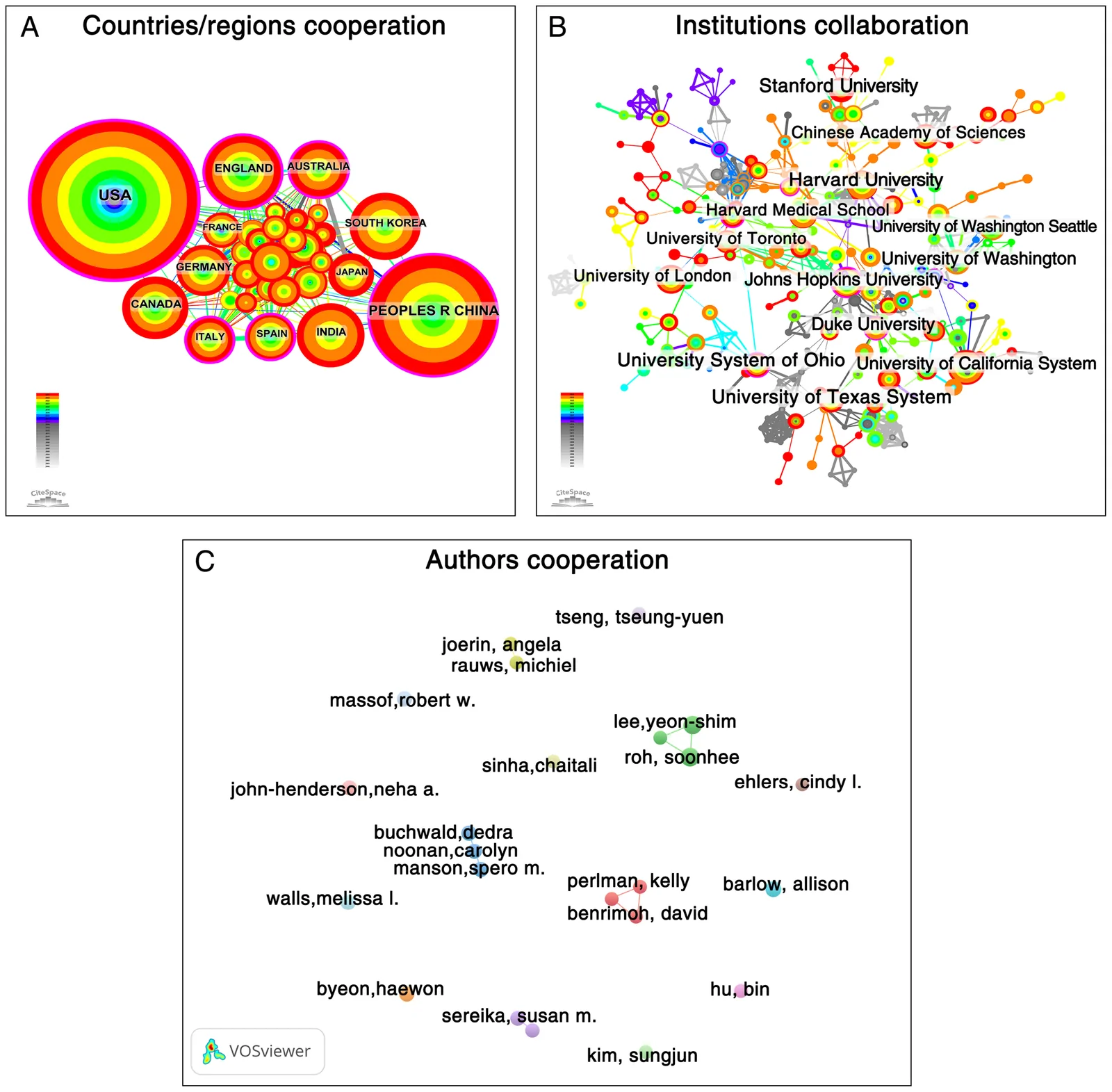
The co-occurrence map of (A) countries/regions, (B) institutions, (C) and authors in AI and depression research. AI = artificial intelligence.

As shown in Figure 3, the large number of nodes and the extensive connections indicate close scientific collaboration across 3 dimensions: countries, institutions, and authors. The country collaboration network consists of 85 nodes and 552 connections, with nodes representing countries such as the United States, China, the United Kingdom, Canada, and South Korea, as shown in Figure 3A. The institution collaboration network includes 454 nodes and 718 connections, with the leading institutions being the University of California System, Harvard University, Johns Hopkins University, the University of London, and the University of Washington, as illustrated in Figure 3B. The author collaboration map is shown in Figure 3C. The papers by Lee, Yeon-Shim; Roh, Soonhee; Barlow, Allison; Buchwald, Dedra; and John-Henderson, Neha A. have the highest number of publications in the field. The dense connections represent extensive research collaboration among the authors. Notably, a clustering effect is observed among the nodes of authors Lee, Yeon-Shim and Roh, Soonhee, with these nodes forming a cluster. Similarly, nodes such as Buchwald, Dedra, Manson, Spero M., and Noonan, Carolyn form another cluster.

### 3.2. Variation of the most active topics

#### 3.2.1. Subject category burst

**Figure 4.**
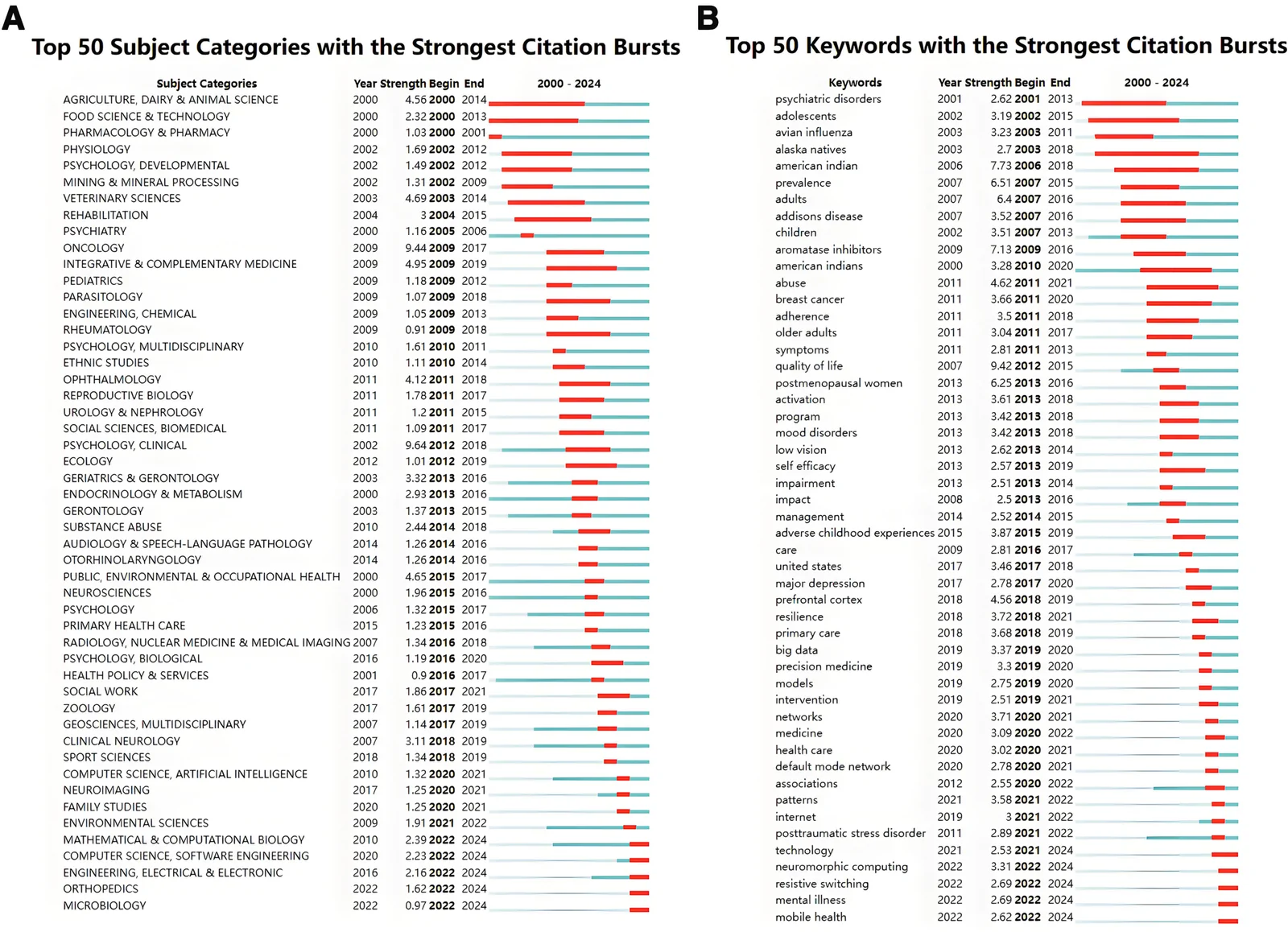
Top 50 (A) subject categories (B) and keywords with the strongest citation bursts.

From 2000 to 2024, citation bursts were reported in 165 out of a total of 181 relevant disciplines. The blue line represents this time interval, while the red line segment indicates the duration of the surge in subject categories, clearly marking its start and end years. Figure 4A shows the top 50 categories with the highest burst intensity during different time periods. The burst period for the subject category Agriculture, Dairy & Animal Science occurred between 2000 and 2014, with the longest duration of burst intensity, and the burst intensity was 4.56. Notably, over time, the “burst” phenomenon in subject categories has gradually diversified, as seen in categories such as Psychology, Developmental (2002–2012), Rehabilitation (2004–2015), Integrative & Complementary Medicine (2009–2019), Reproductive Biology (2011–2017), Health Policy & Services (2016–2017), Computer Science, Artificial Intelligence (2020–2021), and Microbiology (2022–2024). The changes in burst disciplines along the timeline indicate the multidisciplinary nature of the field. Furthermore, beginning in 2024, there will be a total of 20 burst disciplines (see Table S2, Supplemental Digital Content 2 online). The top 3 disciplines are Computer Science, Information Systems (2023–2024), Biology (2023–2024), and Mathematical & Computational Biology (2022–2024).

#### 3.2.2. Keywords burst

At a more granular level, the active content of AI in the field of depression over the entire time span (2000–2024) is revealed by detecting the burst patterns of keywords.^[21]^ A total of 669 keywords exhibited burst activity at different time points, with the top 50 keywords showing the highest burst intensities displayed in Figure 4B. For instance, the keyword “quality of life” experienced a burst between 2012 and 2015, with its peak burst intensity reaching 9.42. Similarly, the keyword “American Indian” showed a burst between 2006 and 2018, with a burst intensity of 7.73, while the keyword “aromatase inhibitors” had a burst between 2009 and 2016, reaching an intensity of 7.13. Furthermore, we paid particular attention to 35 keywords that continued to emerge in 2023, as they may indicate potential research hotspots in the coming years. For example, the burst intensity for “depression detection” between 2023 and 2024 is 3.62, while “natural language processing” has a burst intensity of 3.4 over the same period. Other notable emerging topics include “neuromorphic computing” (burst intensity 3.31, 2022–2024) and “explainable AI” (burst intensity 3.29, 2023–2024) (see Table S2, Supplemental Digital Content 2 online).

#### 3.2.3. Reference burst

After analysis, a total of 251 burst articles were identified. Table S3, Supplemental Digital Content 3 lists the top 30 most cited references from 2000 to 2024 (see Table S3, Supplemental Digital Content 3 online). The article titled “Delivering Cognitive Behavior Therapy to Young Adults with Symptoms of Depression and Anxiety Using a Fully Automated Conversational Agent (Woebot): A Randomized Controlled Trial” has the highest citation burst rate, with its level of attention remaining high from 2018 to 2022.^[22]^ The article titled “A Nonvolatile Organic Electrochemical Device as a Low-Voltage Artificial Synapse for Neuromorphic Computing” demonstrates a citation impact of 29.63 from 2018 to 2022.^[23]^ The article titled “Cross-trial Prediction of Treatment Outcome in Depression: A Machine Learning Approach” is the third most cited article based on citation burst. Upon its publication, it garnered widespread attention, maintaining a high level of interest over a period of 4 years, from 2018 to 2022.^[24]^

Starting from 2024, there are a total of 65 high-impact articles, with the top 20 articles ranked by strength index shown in Table S4, Supplemental Digital Content 4 (see Table S4, Supplemental Digital Content 4 online). Among them, 4 are “reviews” and 16 are “articles.” All of these papers entered the citation surge period as soon as they were published, either in the same year or the following year. The “review” provides valuable guidance for research on AI in depression, while the “article” offers significant reference value for its application. This also reminds researchers working on AI in depression to pay more attention to this information.

### 3.3. Emerging trends and new developments

#### 3.3.1. The temporal variation of keyword clusters

**Figure 5.**
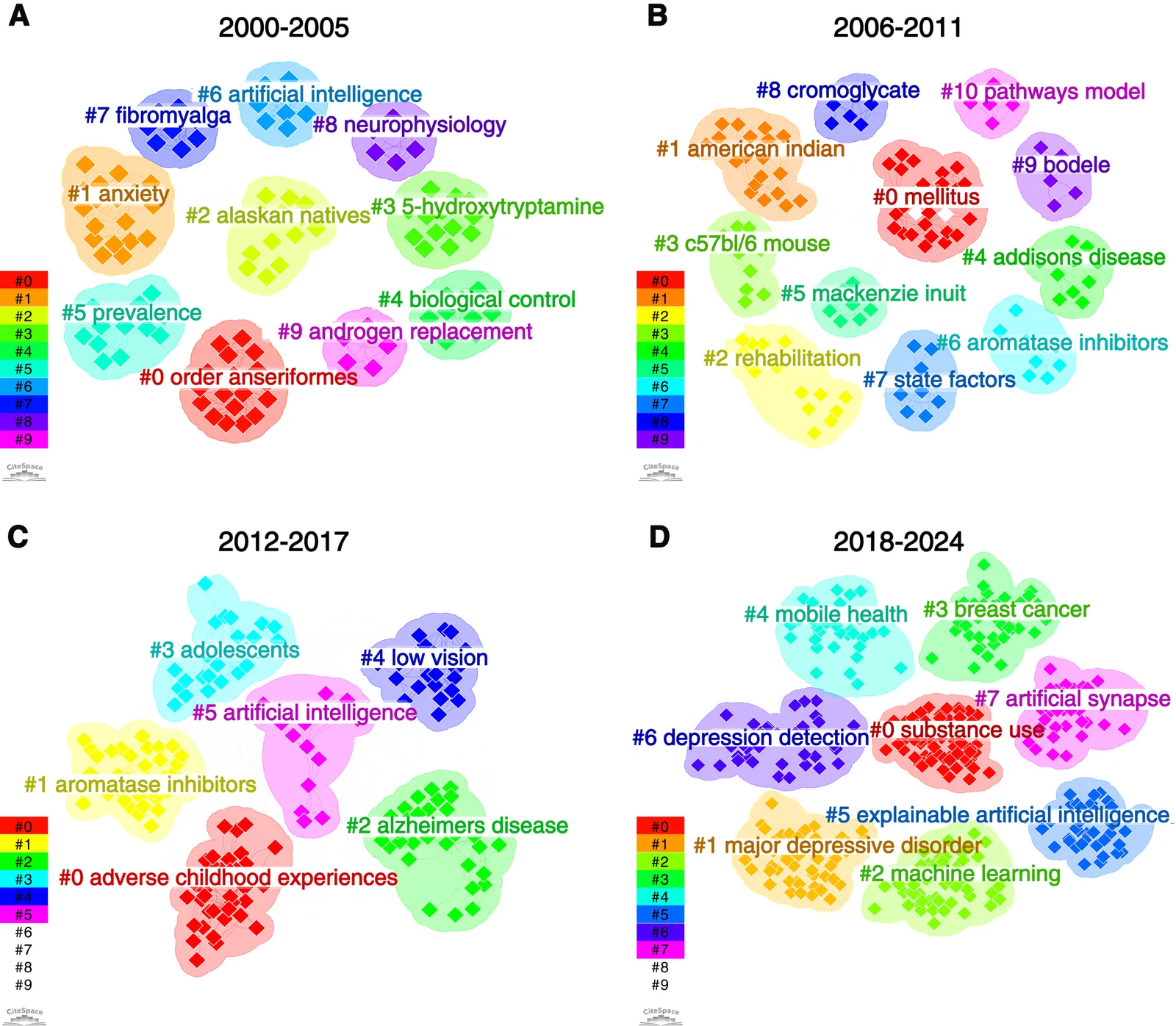
Keyword cluster graph from (A) 2000 to 2005, (B) 2006 to 2011, (C) 2012 to 2017, and (D) 2018 to 2024.

There is a close, inherent connection between keywords, and certain keywords can form different clusters based on their relationships.^[25]^ Identifying these clusters helps to more intuitively outline the various subfields of focus within AI research on depression. The nearly 20-year period is divided into 4 stages, each lasting 6 years. The keyword clusters for each stage are shown in Figure 5. The first clustering analysis (2000–2005) was based on 31 papers, resulting in 10 clusters, including #0 order Anseriformes, #1 anxiety, #2 Alaskan natives, and others (Fig. 5A). The second clustering analysis (2006–2011) was based on 51 papers, resulting in 11 clusters, including #0 mellitus, #1 American Indian, #2 rehabilitation, and others (Fig. 5B). The third clustering analysis (2012–2017) was conducted on 466 papers, resulting in 6 clusters, including #0 adverse childhood experiences, #1 aromatase inhibitors, #2 Alzheimer’s disease, and others (Fig. 5C). In the fourth clustering analysis (2018–2024), a total of 1210 papers were included, resulting in 8 clusters, namely #0 substance use, #1 major depressive disorder, #2 machine learning, among others (Fig. 5D). Compared to the previous 15 years, classic research topics, such as explainable AI, continue to be prominent, while emerging research clusters, such as #0 substance use, #1 major depressive disorder, #2 machine learning, #3 breast cancer, #4 mobile health, #6 depression detection, and #7 artificial synapse, have garnered increased attention from researchers.

Our interpretation of the emerging cluster literature indicates that these clusters primarily aim to explore various aspects of AI in depression research. Cluster #0, “Substance use,” includes 72 articles related to the application of AI in the context of depression. Cluster #1, “Major depressive disorder,” and Cluster #6, “Depression detection,” collected 55 and 36 articles, respectively, focusing on the use of AI in the diagnosis of depression. Cluster #2, “Machine learning,” encompasses 54 articles on the use of ML and big data in depression research. Cluster #3, “Breast cancer,” includes 40 articles discussing depression during endocrine therapy for breast cancer. Cluster #4, “Mobile health,” also gathered 40 articles, while Cluster #7, “Artificial synapse,” collected 34 articles related to artificial synapses.

Table S5, Supplemental Digital Content 5 shows the detailed data for the fourth cluster (2018–2024) (see Table S5, Supplemental Digital Content 5 online). The representative keywords within these clusters help to identify the core research areas of AI in depression during the most recent period (2018–2024).

#### 3.3.2. The keyword alluvial flow visualization

**Figure 6.**
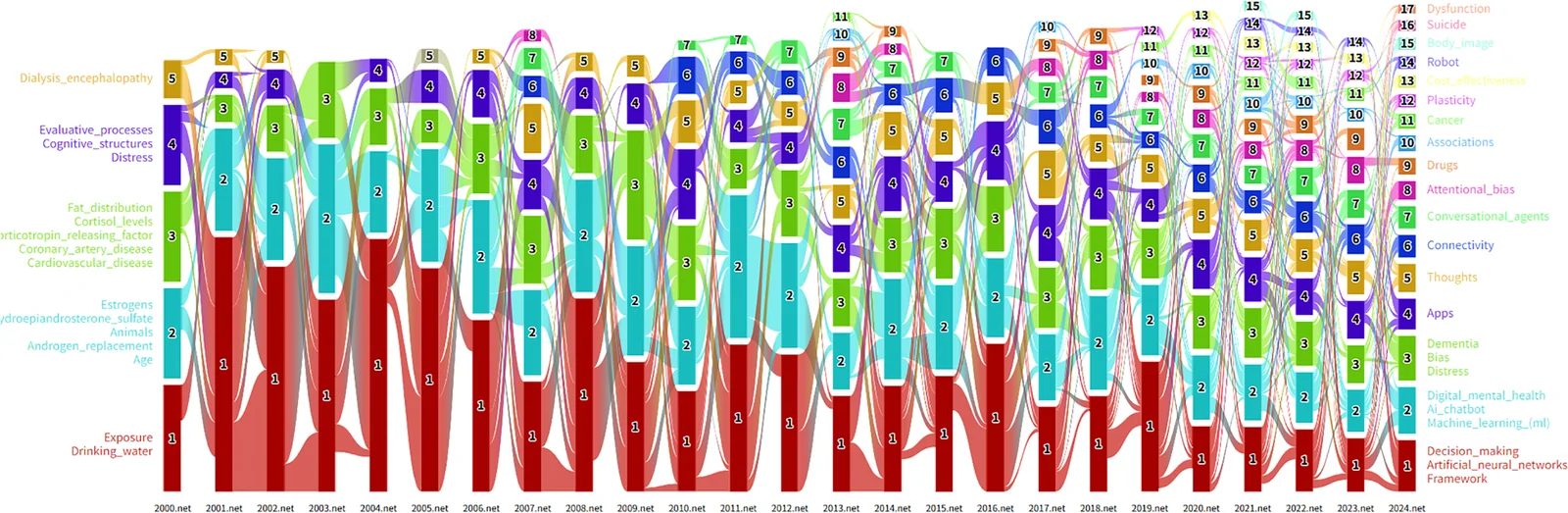
2000 to 2024 keyword alluvial flow visualization, X-axis: time, Y-axis: modules.

As shown in Figure 6, associated keywords can be combined into specific research modules. As these keywords are recombined, the research modules may either differentiate or aggregate over time, resulting in the formation of new modules.

Over the past 25 years, certain high-traffic keywords have demonstrated significant vitality; some have evolved into new research trends, while others have gradually faded from the historical trajectory of the field.^[26]^ Table S6, Supplemental Digital Content 6 lists the most frequently occurring keywords for the top 5 modules each year (see Table S6, Supplemental Digital Content 6 online).

**Figure 7.**
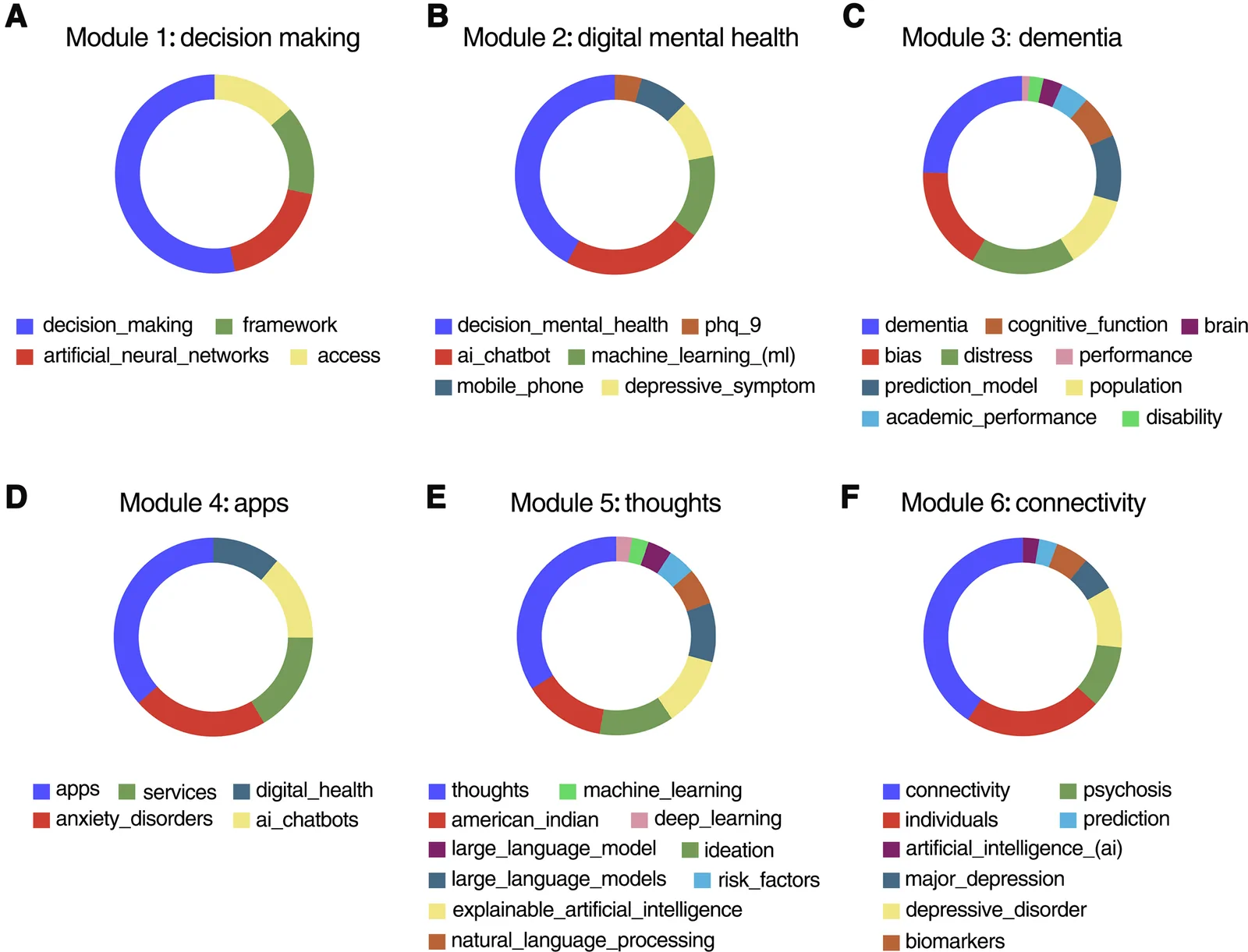
Keywords of the top 6 modules in 2024. (A) Module 1 (decision-making), (B) Module 2 (digital mental health), (C) Module 3 (dementia), (D) Module 4 (apps), (E) Module 5 (thoughts), (F) Module 6 (connectivity).

Clearly, the keywords included in Module 1 of 2024 either diverge or converge within this research domain, thereby forming the largest research tributary (highlighted in red). This suggests that Module 1 is the most enduring research module. Additionally, we have also plotted all the keywords of the top 6 modules in 2024 (Fig. 7). Module 1 is named “decision making” and includes 4 keywords, such as artificial neural networks, framework, access, and others (Fig. 7A). Module 2 is named “digital mental health” and includes 6 keywords, such as AI chatbot, ML, depressive symptom, and mobile phone, and others (Fig. 7B). Module 3 is named “dementia” and includes ten keywords, such as bias, distress, population, and prediction model, and others (Fig. 7C). Module 4 is named “apps” and includes 5 keywords, such as anxiety disorders, services, and AI chatbots, and others (Fig. 7D). Module 5 is named “thoughts” and includes ten keywords, such as American Indian, ideation, explainable AI, large language models, and others (Fig. 7E). Module 6 is named “connectivity” and includes 8 keywords, such as individuals, psychosis, depressive disorder, major depression, biomarkers, and others (Fig. 7F). These modules may represent emerging trends in the field of depression for AI over the next 5 years or even longer.

#### 3.3.3. The timeline visualization of references

**Figure 8.**
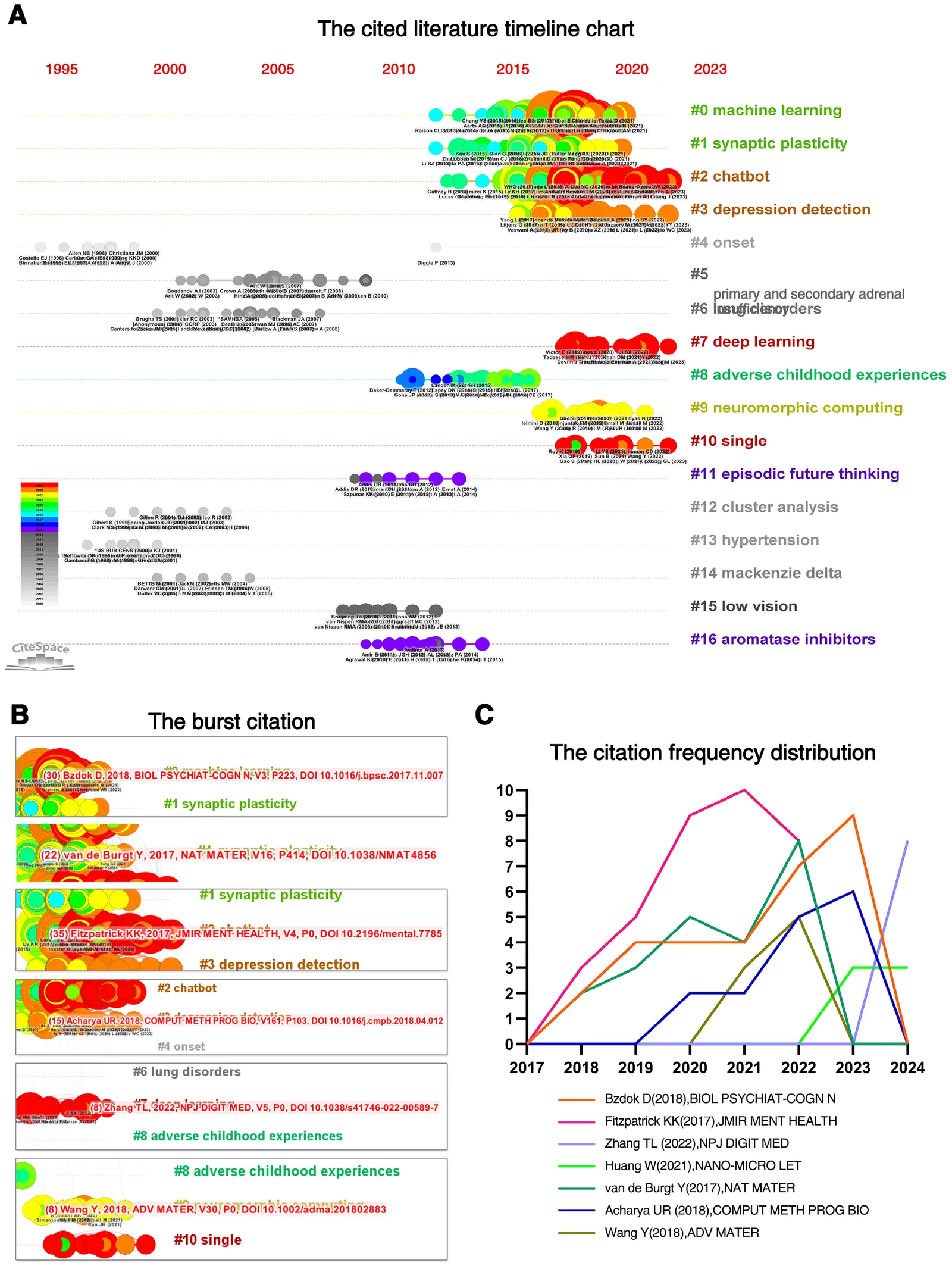
The reference clusters map. (A) The cited literature timeline chart, (B) the burst citation in #0, #1, #2, #3, #7, #9, and #10, and (C) the citation frequency distribution of the burst citation in #0, #1, #2, #3, #7, #9, and #10, X-axis: year, Y-axis: cited frequency.

The timeline visualization, based on the citation time span, can predict which topics are emerging, which are classic, and which are relatively outdated.^[27]^ The timeline chart of AI in depression research consists of 16 groups at a given time, with the groups arranged from top to bottom according to size (Fig. 8A). Among them, topics #8 adverse childhood experiences, #11 episodic future thinking, #15 low vision, and #16 aromatase inhibitors are considered classic. While these topics may not be the most recent, they are closely connected with other groups. In contrast, topics #4 onset, #5 primary and secondary adrenal insufficiency, #6 lung disorders, #12 cluster analysis, #13 hypertension, and #14 Mackenzie delta are relatively outdated. These groups have limited connections with others and exhibit no subsequent developments within their own timelines. Finally, topics #0 machine learning, #1 synaptic plasticity, #2 chatbot, #3 depression detection, #7 deep learning, #9 neuromorphic computing, and #10 single are emerging topics, as they have remained active on the timeline since their inception. This suggests that these fields are likely to become key areas of research in the future. Table S7, Supplemental Digital Content 7 provides a more detailed overview of the emerging topics (see Table S7, Supplemental Digital Content 7 online). Additionally, some seminal papers (represented by the large nodes marked with red circles) have played a pivotal role in advancing the development of these subfields (Fig. 8B).

The article by Bzdok, Danilo, published in 2018, belongs to Group #0, with a total of 30 co-citations.^[28]^ The article “A nonvolatile organic electrochemical device as a low-voltage artificial synapse for neuromorphic computing” by van de Burgt, Yoeri, belongs to Group #1, with a co-occurrence frequency of 22.^[23]^ The article “Delivering Cognitive Behavior Therapy to Young Adults with Symptoms of Depression and Anxiety Using a Fully Automated Conversational Agent (Woebot): A Randomized Controlled Trial” by Fitzpatrick Kathleen Kara, belongs to Group #2, with a co-occurrence frequency of 35.^[22]^ The article “Automated EEG-based screening of depression using deep convolutional neural network,” published in Computer Methods and Programs in Biomedicine, belongs to Group #3, with a co-occurrence frequency of 15.^[29]^ The article “Natural language processing applied to mental illness detection: a narrative review” belongs to Group #7, with a co-occurrence frequency of 8.^[30]^ The article “Photonic Synapses Based on Inorganic Perovskite Quantum Dots for Neuromorphic Computing” belongs to Group #9, with a co-occurrence frequency of 8.^[31]^ The article “Memristive Artificial Synapses for Neuromorphic Computing” belongs to Group #10, with a co-occurrence frequency of 6.^[32]^ We further analyzed the citation distribution of these 7 articles over recent years (Fig. 8C), which allows us to predict the likelihood that these articles will be cited again in the coming years.

## 4. Discussion

In this study, we conducted a bibliometric analysis to explore the evolving relationship between depression and AI research. By examining a comprehensive set of relevant publications from leading journals, we identified significant trends, key developments, and emerging research topics within this interdisciplinary field. Our findings not only highlight the increasing academic interest in the application of AI to the study and treatment of depression but also reveal the growing integration of ML, deep learning, and other AI technologies in advancing mental health diagnostics and interventions.

### 4.1. Emergent trends and research focus

One of the most prominent trends identified in this study is the rapid increase in publications that intersect AI and depression over the past 6 years. Early studies primarily focused on exploring the basic capabilities of AI models in mental health applications, with an emphasis on algorithmic accuracy and prediction models for depression diagnosis.^[33]^ However, more recent works have shifted focus to the nuanced aspects of this intersection, such as personalized treatment models, AI-driven cognitive behavioral therapy, and predictive modeling for depression relapse.^[5,34]^ This shift suggests that AI is moving beyond merely supporting diagnostic functions to actively shaping therapeutic strategies for depression, offering personalized interventions tailored to individual patients.

Moreover, our analysis reveals a substantial increase in the collaboration between computer science, psychiatry, and neuroscience researchers. These interdisciplinary collaborations are essential for bridging the knowledge gaps between the technical capabilities of AI and the complex, multifactorial nature of depression.^[35]^ The application of AI in the context of mental health disorders has the potential to uncover novel biomarkers, predict treatment responses, and provide real-time data for clinicians.^[36]^ However, as the field progresses, it is imperative to ensure that AI models are rigorously validated, through robust clinical trials and cross-validation studies, to confirm their real-world applicability and minimize risks such as algorithmic bias.

### 4.2. The role of ML and deep learning

A key observation in our bibliometric analysis is the increasing prevalence of ML and deep learning techniques in depression research.^[37]^ Early studies, which employed traditional statistical models, have progressively been replaced by more sophisticated approaches, including neural networks, reinforcement learning, and natural language processing.^[38]^ These advanced techniques are particularly useful in analyzing large, complex datasets, such as electronic health records, social media data, and neuroimaging data, which are increasingly being utilized to understand the etiology of depression and predict patient outcomes.^[39]^

AI-driven models, particularly deep learning algorithms, have shown great promise in processing high-dimensional data, enabling the identification of hidden patterns in patient behavior and brain activity.^[40]^ For instance, recent studies have demonstrated the ability of AI to classify depression subtypes based on brain imaging and genetic data, offering insights into the heterogeneity of depression and facilitating more targeted therapeutic interventions.^[41,42]^ Additionally, natural language processing techniques are being used to analyze patient conversations, social media posts, and digital footprints to detect early signs of depression and monitor patient progress over time.^[43]^

### 4.3. Ethical and practical implications

While the potential for AI in depression research and treatment is undeniable, significant ethical and practical concerns must be addressed. First and foremost, the issue of data privacy and patient confidentiality is paramount, especially when dealing with sensitive information such as mental health records and personal behavioral data.^[44]^ Ensuring that AI systems comply with data protection regulations, such as General Data Protection Regulation or Health Insurance Portability and Accountability Act, is crucial to maintaining trust between patients and healthcare providers.^[45]^

Additionally, the risk of algorithmic bias must be carefully considered. AI systems are only as good as the data on which they are trained, and biased data can lead to biased outcomes, potentially exacerbating health disparities.^[46]^ For example, an AI model trained predominantly on data from 1 demographic group may not generalize well to others, resulting in disparities in diagnosis and treatment. Therefore, it is essential for future research to focus on creating more diverse, representative datasets to train AI models and ensure that AI-driven interventions are equitable and inclusive.^[47]^

Finally, the integration of AI in clinical settings presents practical challenges related to its acceptance by healthcare professionals and patients. Despite the growing body of evidence supporting the efficacy of AI-based interventions, there remains a degree of skepticism and reluctance to adopt AI technologies in routine clinical practice.^[48]^ This barrier can be mitigated through targeted educational initiatives and pilot programs that demonstrate the benefits of AI in enhancing patient care while preserving the human aspects of the clinician-patient relationship.^[49]^

### 4.4. Limitations

Inevitably, this bibliometric study has several limitations. First, there is the potential incomprehensiveness of our search strategy for specific subtypes of depression. Although our search terms (“depression,” “depressive symptoms,” etc) were designed to capture as widely as possible the literature related to AI and depression, the strategy did not specifically focus on specific, less common index subtypes such as “dysthymia.” These studies may be underestimated or missed. Second, the study only searched the Web of Science database, which is the most influential multidisciplinary academic literature abstract index database worldwide. However, bibliometric analysis using multiple data sources such as PsycINFO, Scopus, and PubMed will be more comprehensive. Finally, the field of AI is rapidly evolving, with new research on its application in depression published monthly. Thus, the results of this study can only reflect the current state of the art.

### 4.5. Future directions

Looking ahead, several key areas of research warrant further exploration. First, there is a need for more longitudinal studies to assess the long-term effects of AI-driven treatments for depression.^[50]^ While short-term outcomes are promising, the long-term efficacy, safety, and sustainability of these treatments remain unclear.^[51]^ Second, as AI models continue to evolve, there is an increasing need for standardization and transparency in AI algorithms used in clinical settings. Establishing clear guidelines for the development, validation, and implementation of AI-based tools will be essential for their widespread acceptance and use in clinical practice.^[52]^

Finally, interdisciplinary research that combines AI with emerging fields such as neuroplasticity, psychopharmacology, and digital mental health is likely to yield novel insights into the underlying mechanisms of depression and improve treatment outcomes.^[53]^ By fostering collaboration across disciplines, we can ensure that AI plays a transformative role in advancing our understanding of depression and enhancing the quality of care for individuals affected by this debilitating disorder.^[54]^

## 5. Conclusion

In conclusion, our bibliometric analysis demonstrates that the intersection of depression and AI is a rapidly growing area of research with significant potential to revolutionize mental healthcare. As AI technologies continue to evolve, they hold promise not only for improving diagnostic accuracy and treatment outcomes but also for offering more personalized, scalable, and accessible interventions for depression. However, it is essential for future research to address ethical, practical, and methodological challenges to ensure that AI is deployed in a way that maximizes its potential benefits while minimizing risks. With continued interdisciplinary collaboration and careful validation, AI has the potential to become a key tool in the fight against depression, ultimately improving the lives of millions of people worldwide.

## Acknowledgments

The authors sincerely appreciate the editors and anonymous reviewers for their insightful feedback and constructive suggestions, which have significantly improved the quality of this paper.

## Author contributions

**Conceptualization:** Chunrong He, Junkang Zhao.

**Data curation:** Chunrong He.

**Formal analysis:** Chunrong He, Junkang Zhao.

**Funding acquisition:** Chunrong He, Junkang Zhao.

**Investigation:** Chunrong He.

**Methodology:** Chunrong He, Junkang Zhao.

**Project administration:** Chunrong He, Junkang Zhao.

**Resources:** Chunrong He, Junkang Zhao.

**Supervision:** Chunrong He, Junkang Zhao.

**Validation:** Chunrong He, Junkang Zhao.

**Visualization:** Chunrong He, Junkang Zhao.

**Writing – original draft:** Chunrong He

**Writing – review & editing:** Chunrong He, Junkang Zhao.














