# Supplementary material for: Research on the correlation between artificial intelligence and depression: A bibliometric analysis from 2000 to 2024
Source: Medicine (Baltimore). 2026 Sep 18;105(38):e50781. doi: 10.1097/MD.0000000000050781 (PMC13593024; doi:10.1097/MD.0000000000050781)
Supplement: Supplementary file 1 [file medi-105-e50781-s001.docx]

**TABLE S1** Top 5 articles in centrality.

| NO | Article information | Journal | LCS | GCS |
| --- | --- | --- | --- | --- |
| 1 | Applications of machine learning algorithms to predict therapeutic outcomes in depression: A meta-analysis and systematic review | J AFFECT DISORDERS | 18 | 158 |
| 2 | Conversational agents in healthcare: a systematic review | J AM MED INFORM ASSN | 17 | 478 |
| 3 | Tunable synaptic behavior realized in C3N composite based memristor | NANO ENERGY | 10 | 127 |
| 4 | Artificially intelligent chatbots in digital mental health interventions: a review | EXPERT REV MED DEVIC | 9 | 72 |
| 5 | Automated depression analysis using convolutional neural networks from speech | J BIOMED INFORM | 8 | 70 |

***Note.*** *LCS: Local Citation Score, GCS: Global Citation Score.*
