# Supplementary material for: Research on the correlation between artificial intelligence and depression: A bibliometric analysis from 2000 to 2024
Source: Medicine (Baltimore). 2026 Sep 18;105(38):e50781. doi: 10.1097/MD.0000000000050781 (PMC13593024; doi:10.1097/MD.0000000000050781)
Supplement: Supplementary file 2 [file medi-105-e50781-s002.docx]

**TABLE S2**. The top 20 subject categories and keywords burst with a burst period from beginning to 2024.

| **Subject category bursts** | | | | | **Keywords bursts** | | | | |
| --- | --- | --- | --- | --- | --- | --- | --- | --- | --- |
| Begin | End | Strength | Year | Entity | Begin | End | Strength | Year | Entity |
| 2023 | 2024 | 3.79 | 2015 | COMPUTER SCIENCE, INFORMATION SYSTEMS | 2023 | 2024 | 3.62 | 2023 | depression detection |
| 2023 | 2024 | 2.76 | 2019 | BIOLOGY | 2023 | 2024 | 3.4 | 2020 | natural language processing |
| 2022 | 2024 | 2.39 | 2010 | MATHEMATICAL & COMPUTATIONAL BIOLOGY | 2022 | 2024 | 3.31 | 2022 | neuromorphic computing |
| 2022 | 2024 | 2.23 | 2020 | COMPUTER SCIENCE, SOFTWARE ENGINEERING | 2023 | 2024 | 3.29 | 2023 | explainable ai |
| 2022 | 2024 | 2.16 | 2016 | ENGINEERING, ELECTRICAL & ELECTRONIC | 2023 | 2024 | 2.96 | 2023 | phq 9 |
| 2023 | 2024 | 1.79 | 2019 | COMPUTER SCIENCE, CYBERNETICS | 2022 | 2024 | 2.69 | 2022 | mental illness |
| 2023 | 2024 | 1.72 | 2022 | MATHEMATICS | 2022 | 2024 | 2.69 | 2022 | resistive switching |
| 2023 | 2024 | 1.7 | 2017 | ENGINEERING, MULTIDISCIPLINARY | 2023 | 2024 | 2.68 | 2019 | performance |
| 2023 | 2024 | 1.65 | 2012 | MULTIDISCIPLINARY SCIENCES | 2023 | 2024 | 2.63 | 2023 | postpartum depression |
| 2022 | 2024 | 1.62 | 2022 | ORTHOPEDICS | 2022 | 2024 | 2.62 | 2022 | mobile health |
| 2022 | 2024 | 0.97 | 2022 | MICROBIOLOGY | 2021 | 2024 | 2.53 | 2021 | technology |
| 2023 | 2024 | 0.82 | 2021 | ENVIRONMENTAL STUDIES | 2023 | 2024 | 2.45 | 2023 | states |
| 2023 | 2024 | 0.79 | 2020 | CHEMISTRY, ANALYTICAL | 2023 | 2024 | 2.45 | 2023 | covid 19 |
| 2023 | 2024 | 0.75 | 2020 | DENTISTRY, ORAL SURGERY & MEDICINE | 2023 | 2024 | 2.45 | 2023 | parkinsons disease |
| 2023 | 2024 | 0.61 | 2023 | ERGONOMICS | 2023 | 2024 | 2.34 | 2021 | feature selection |
| 2023 | 2024 | 0.61 | 2023 | GEOLOGY | 2023 | 2024 | 2.3 | 2023 | onset |
| 2023 | 2024 | 0.61 | 2023 | MATERIALS SCIENCE, CERAMICS | 2023 | 2024 | 2.3 | 2023 | information |
| 2023 | 2024 | 0.6 | 2021 | GREEN & SUSTAINABLE SCIENCE & TECHNOLOGY | 2022 | 2024 | 2.26 | 2022 | system |
| 2021 | 2024 | 0.59 | 2021 | SOCIAL ISSUES | 2022 | 2024 | 2.22 | 2018 | efficacy |
| 2023 | 2024 | 0.58 | 2018 | INSTRUMENTS & INSTRUMENTATION | 2023 | 2024 | 2.2 | 2020 | neural network |

***Note.*** *Begin: the burst’ beginning year, End: the burst’ ending year, Strength: the burst’ strength index, Year: the first appearance time, Entity: the term.*
