# Supplementary material for: Research on the correlation between artificial intelligence and depression: A bibliometric analysis from 2000 to 2024
Source: Medicine (Baltimore). 2026 Sep 18;105(38):e50781. doi: 10.1097/MD.0000000000050781 (PMC13593024; doi:10.1097/MD.0000000000050781)
Supplement: Supplementary file 3 [file medi-105-e50781-s003.docx]

**TABLE S3** The references with citation bursts at different period.

| **References** | **Year** | **Strength** | **Begin** | **End** | **2004 - 2024** |
| --- | --- | --- | --- | --- | --- |
| Gone JP, 2012, ANNU REV CLIN PSYCHO, V8, P131, DOI 10.1146/annurev-clinpsy-032511-143127, [DOI](http://dx.doi.org/10.1146%2Fannurev-clinpsy-032511-143127) | 2012 | 4.02 | **2016** | 2017 | ▂▂▂▂▂▂▂▂▂▂▂▂▂▂▂▂▃▃▂▂▂▂▂▂▂ |
| Fitzpatrick KK, 2017, JMIR MENT HEALTH, V4, P0, DOI 10.2196/mental.7785, [DOI](http://dx.doi.org/10.2196%2Fmental.7785) | 2017 | 8.24 | **2018** | 2022 | ▂▂▂▂▂▂▂▂▂▂▂▂▂▂▂▂▂▂▃▃▃▃▃▂▂ |
| van de Burgt Y, 2017, NAT MATER, V16, P414, DOI 10.1038/NMAT4856, [DOI](http://dx.doi.org/10.1038%2FNMAT4856) | 2017 | 5.14 | **2018** | 2022 | ▂▂▂▂▂▂▂▂▂▂▂▂▂▂▂▂▂▂▃▃▃▃▃▂▂ |
| Wang ZR, 2017, NAT MATER, V16, P101, DOI 10.1038/nmat4756, [DOI](http://dx.doi.org/10.1038%2Fnmat4756) | 2017 | 4.67 | **2018** | 2022 | ▂▂▂▂▂▂▂▂▂▂▂▂▂▂▂▂▂▂▃▃▃▃▃▂▂ |
| Zhong YN, 2018, ADV FUNCT MATER, V28, P0, DOI 10.1002/adfm.201800854, [DOI](http://dx.doi.org/10.1002%2Fadfm.201800854) | 2018 | 4.15 | **2018** | 2019 | ▂▂▂▂▂▂▂▂▂▂▂▂▂▂▂▂▂▂▃▃▂▂▂▂▂ |
| John RA, 2018, ADV MATER, V30, P0, DOI 10.1002/adma.201800220, [DOI](http://dx.doi.org/10.1002%2Fadma.201800220) | 2018 | 3.95 | **2018** | 2020 | ▂▂▂▂▂▂▂▂▂▂▂▂▂▂▂▂▂▂▃▃▃▂▂▂▂ |
| Zhu LQ, 2014, NAT COMMUN, V5, P0, DOI 10.1038/ncomms4158, [DOI](http://dx.doi.org/10.1038%2Fncomms4158) | 2014 | 2.96 | **2018** | 2019 | ▂▂▂▂▂▂▂▂▂▂▂▂▂▂▂▂▂▂▃▃▂▂▂▂▂ |
| Esteva A, 2017, NATURE, V542, P115, DOI 10.1038/nature21056, [DOI](http://dx.doi.org/10.1038%2Fnature21056) | 2017 | 4.09 | **2019** | 2021 | ▂▂▂▂▂▂▂▂▂▂▂▂▂▂▂▂▂▂▂▃▃▃▂▂▂ |
| Chekroud AM, 2016, LANCET PSYCHIAT, V3, P243, DOI 10.1016/S2215-0366(15)00471-X, [DOI](http://dx.doi.org/10.1016%2FS2215-0366%2815%2900471-X) | 2016 | 4.09 | **2019** | 2021 | ▂▂▂▂▂▂▂▂▂▂▂▂▂▂▂▂▂▂▂▃▃▃▂▂▂ |
| Ly KH, 2017, INTERNET INTERV, V10, P39, DOI 10.1016/j.invent.2017.10.002, [DOI](http://dx.doi.org/10.1016%2Fj.invent.2017.10.002) | 2017 | 3.68 | **2019** | 2021 | ▂▂▂▂▂▂▂▂▂▂▂▂▂▂▂▂▂▂▂▃▃▃▂▂▂ |
| Jiang F, 2017, STROKE VASC NEUROL, V2, P230, DOI 10.1136/svn-2017-000101, [DOI](http://dx.doi.org/10.1136%2Fsvn-2017-000101) | 2017 | 3.52 | **2019** | 2022 | ▂▂▂▂▂▂▂▂▂▂▂▂▂▂▂▂▂▂▂▃▃▃▃▂▂ |
| Wu C, 2017, NAT COMMUN, V8, P0, DOI 10.1038/s41467-017-00803-1, [DOI](http://dx.doi.org/10.1038%2Fs41467-017-00803-1) | 2017 | 3.27 | **2019** | 2021 | ▂▂▂▂▂▂▂▂▂▂▂▂▂▂▂▂▂▂▂▃▃▃▂▂▂ |
| Jiang J, 2017, SMALL, V13, P0, DOI 10.1002/smll.201700933, [DOI](http://dx.doi.org/10.1002%2Fsmll.201700933) | 2017 | 3.25 | **2019** | 2022 | ▂▂▂▂▂▂▂▂▂▂▂▂▂▂▂▂▂▂▂▃▃▃▃▂▂ |
| Liu YH, 2015, ADV MATER, V27, P5599, DOI 10.1002/adma.201502719, [DOI](http://dx.doi.org/10.1002%2Fadma.201502719) | 2015 | 3.19 | **2019** | 2020 | ▂▂▂▂▂▂▂▂▂▂▂▂▂▂▂▂▂▂▂▃▃▂▂▂▂ |
| Onnela JP, 2016, NEUROPSYCHOPHARMACOL, V41, P1691, DOI 10.1038/npp.2016.7, [DOI](http://dx.doi.org/10.1038%2Fnpp.2016.7) | 2016 | 3.78 | **2020** | 2021 | ▂▂▂▂▂▂▂▂▂▂▂▂▂▂▂▂▂▂▂▂▃▃▂▂▂ |
| Obermeyer Z, 2019, SCIENCE, V366, P447, DOI 10.1126/science.aax2342, [DOI](http://dx.doi.org/10.1126%2Fscience.aax2342) | 2019 | 3.35 | **2020** | 2022 | ▂▂▂▂▂▂▂▂▂▂▂▂▂▂▂▂▂▂▂▂▃▃▃▂▂ |
| Inkster B, 2018, JMIR MHEALTH UHEALTH, V6, P0, DOI 10.2196/12106, [DOI](http://dx.doi.org/10.2196%2F12106) | 2018 | 3.33 | **2020** | 2024 | ▂▂▂▂▂▂▂▂▂▂▂▂▂▂▂▂▂▂▂▂▃▃▃▃▃ |
| Zhou FC, 2019, NAT NANOTECHNOL, V14, P776, DOI 10.1038/s41565-019-0501-3, [DOI](http://dx.doi.org/10.1038%2Fs41565-019-0501-3) | 2019 | 3.31 | **2020** | 2021 | ▂▂▂▂▂▂▂▂▂▂▂▂▂▂▂▂▂▂▂▂▃▃▂▂▂ |
| Yang JT, 2018, ADV MATER, V30, P0, DOI 10.1002/adma.201801548, [DOI](http://dx.doi.org/10.1002%2Fadma.201801548) | 2018 | 3.31 | **2020** | 2021 | ▂▂▂▂▂▂▂▂▂▂▂▂▂▂▂▂▂▂▂▂▃▃▂▂▂ |
| Dwyer DB, 2018, ANNU REV CLIN PSYCHO, V14, P91, DOI 10.1146/annurev-clinpsy-032816045037, [DOI](http://dx.doi.org/10.1146%2Fannurev-clinpsy-032816045037) | 2018 | 3.3 | **2020** | 2024 | ▂▂▂▂▂▂▂▂▂▂▂▂▂▂▂▂▂▂▂▂▃▃▃▃▃ |
| Fulmer R, 2018, JMIR MENT HEALTH, V5, P0, DOI 10.2196/mental.9782, [DOI](http://dx.doi.org/10.2196%2Fmental.9782) | 2018 | 3.27 | **2020** | 2021 | ▂▂▂▂▂▂▂▂▂▂▂▂▂▂▂▂▂▂▂▂▃▃▂▂▂ |
| Vaswani A, 2017, ADV NEUR IN, V30, P0 | 2017 | 3.48 | **2021** | 2022 | ▂▂▂▂▂▂▂▂▂▂▂▂▂▂▂▂▂▂▂▂▂▃▃▂▂ |
| Wang Y, 2018, ADV MATER, V30, P0, DOI 10.1002/adma.201802883, [DOI](http://dx.doi.org/10.1002%2Fadma.201802883) | 2018 | 3.48 | **2021** | 2022 | ▂▂▂▂▂▂▂▂▂▂▂▂▂▂▂▂▂▂▂▂▂▃▃▂▂ |
| Baumel A, 2019, J MED INTERNET RES, V21, P0, DOI 10.2196/14567, [DOI](http://dx.doi.org/10.2196%2F14567) | 2019 | 3.04 | **2021** | 2022 | ▂▂▂▂▂▂▂▂▂▂▂▂▂▂▂▂▂▂▂▂▂▃▃▂▂ |
| Lundberg SM, 2017, ADV NEUR IN, V30, P0 | 2017 | 3.04 | **2021** | 2022 | ▂▂▂▂▂▂▂▂▂▂▂▂▂▂▂▂▂▂▂▂▂▃▃▂▂ |
| Graham S, 2019, CURR PSYCHIAT REP, V21, P0, DOI 10.1007/s11920-019-1094-0, [DOI](http://dx.doi.org/10.1007%2Fs11920-019-1094-0) | 2019 | 4.01 | **2022** | 2024 | ▂▂▂▂▂▂▂▂▂▂▂▂▂▂▂▂▂▂▂▂▂▂▃▃▃ |
| Dai SL, 2019, ADV FUNCT MATER, V29, P0, DOI 10.1002/adfm.201903700, [DOI](http://dx.doi.org/10.1002%2Fadfm.201903700) | 2019 | 3.8 | **2022** | 2024 | ▂▂▂▂▂▂▂▂▂▂▂▂▂▂▂▂▂▂▂▂▂▂▃▃▃ |
| Yan XB, 2019, ADV MATER, V31, P0, DOI 10.1002/adma.201805284, [DOI](http://dx.doi.org/10.1002%2Fadma.201805284) | 2019 | 3.42 | **2022** | 2024 | ▂▂▂▂▂▂▂▂▂▂▂▂▂▂▂▂▂▂▂▂▂▂▃▃▃ |
| Santomauro DF, 2021, LANCET, V398, P1700, DOI 10.1016/S0140-6736(21)02143-7, [DOI](http://dx.doi.org/10.1016%2FS0140-6736%2821%2902143-7) | 2021 | 3.27 | **2022** | 2024 | ▂▂▂▂▂▂▂▂▂▂▂▂▂▂▂▂▂▂▂▂▂▂▃▃▃ |
| Chekroud AM, 2021, WORLD PSYCHIATRY, V20, P154, DOI 10.1002/wps.20882, [DOI](http://dx.doi.org/10.1002%2Fwps.20882) | 2021 | 3.04 | **2022** | 2024 | ▂▂▂▂▂▂▂▂▂▂▂▂▂▂▂▂▂▂▂▂▂▂▃▃▃ |
