# Supplementary material for: Research on the correlation between artificial intelligence and depression: A bibliometric analysis from 2000 to 2024
Source: Medicine (Baltimore). 2026 Sep 18;105(38):e50781. doi: 10.1097/MD.0000000000050781 (PMC13593024; doi:10.1097/MD.0000000000050781)
Supplement: Supplementary file 4 [file medi-105-e50781-s004.docx]

**TABLE S4** The references with citation bursts from beginning to 2024.

| **begin** | **end** | **Strength** | **Year** | **Type** | **Title** |
| --- | --- | --- | --- | --- | --- |
| 2023 | 2024 | 4.14 | 2020 | Article | Visually Interpretable Representation Learning for Depression Recognition from Facial Images |
| 2022 | 2024 | 4.01 | 2019 | Review | Artificial Intelligence for Mental Health and Mental Illnesses: an Overview |
| 2022 | 2024 | 3.8 | 2019 | Review | Recent Advances in Transistor-Based Artificial Synapses |
| 2023 | 2024 | 3.72 | 2021 | Article | Artificial Intelligence-Based Chatbot for Anxiety and Depression in University Students: Pilot Randomized Controlled Trial |
| 2023 | 2024 | 3.72 | 2021 | Review | Machine Learning and Natural Language Processing in Mental Health: Systematic Review |
| 2022 | 2024 | 3.42 | 2019 | Article | Self-Assembled Networked PbS Distribution Quantum Dots for Resistive Switching and Artificial Synapse Performance Boost of Memristors |
| 2020 | 2024 | 3.33 | 2018 | Article | An Empathy-Driven, Conversational Artificial Intelligence Agent (Wysa) for Digital Mental Well-Being: Real-World Data Evaluation Mixed-Methods Study |
| 2020 | 2024 | 3.3 | 2018 | Article | Machine Learning Approaches for Clinical Psychology and Psychiatry |
| 2022 | 2024 | 3.27 | 2021 | Article | Global prevalence and burden of depressive and anxiety disorders in 204 countries and territories in 2020 due to the COVID-19 pandemic |
| 2023 | 2024 | 3.1 | 2019 | Article | Automatic Depression Analysis Using Dynamic Facial Appearance Descriptor and Dirichlet Process Fisher Encoding |
| 2023 | 2024 | 3.1 | 2018 | Article | Automated Depression Diagnosis Based on Deep Networks to Encode Facial Appearance and Dynamics |
| 2023 | 2024 | 3.05 | 2018 | Article | Using Psychological Artificial Intelligence (Tess) to Relieve Symptoms of Depression and Anxiety: Randomized Controlled Trial |
| 2022 | 2024 | 3.04 | 2021 | Article | The promise of machine learning in predicting treatment outcomes in psychiatry |
| 2023 | 2024 | 2.97 | 2021 | Article | A Therapeutic Relational Agent for Reducing Problematic Substance Use (Woebot): Development and Usability Study |
| 2022 | 2024 | 2.92 | 2018 | Article | Automated EEG-based screening of depression using deep convolutional neural network |
| 2020 | 2024 | 2.82 | 2019 | Review | High-performance medicine: the convergence of human and artificial intelligence |
| 2021 | 2024 | 2.82 | 2018 | Article | In-memory computing with resistive switching devices |
| 2022 | 2024 | 2.8 | 2019 | Article | Your Robot Therapist Will See You Now: Ethical Implications of Embodied Artificial Intelligence in Psychiatry, Psychology, and Psychotherapy |
| 2023 | 2024 | 2.79 | 2020 | Article | Effectiveness and Safety of Using Chatbots to Improve Mental Health: Systematic Review and Meta-Analysis |
| 2022 | 2024 | 2.66 | 2020 | Article | Controllable analog resistive switching and synaptic characteristics in ZrO2/ZTO bilayer memristive device for neuromorphic systems signaling pathway and apoptosis-related proteins in rats with ulcerative colitis |
