# Supplementary material for: Research on the correlation between artificial intelligence and depression: A bibliometric analysis from 2000 to 2024
Source: Medicine (Baltimore). 2026 Sep 18;105(38):e50781. doi: 10.1097/MD.0000000000050781 (PMC13593024; doi:10.1097/MD.0000000000050781)
Supplement: Supplementary file 5 [file medi-105-e50781-s005.docx]

**TABLE S5.** Summary of keyword clusters for the most recent stage (2018-2024).

| **ClusterID** | **Size** | **Silhouette** | **Average Year** | **Label (LLR)** | **Representative keywords** |
| --- | --- | --- | --- | --- | --- |
| 0 | 72 | 0.62 | 2020 | substance use | artificial intelligence; alzheimers disease; ad diagnosis; explainable artificial intelligence; knowledge base \| mental health; young adult; athletic identity; explainable artificial intelligence; knowledge base |
| 1 | 55 | 0.707 | 2020 | major depressive disorder | artificial intelligence; machine learning; bipolar disorder; memory deficits; repeated maternal separation \| major depressive disorder; functional connectivity; reward network; state functional magnetic resonance; related circuit |
| 2 | 54 | 0.78 | 2020 | machine learning | artificial intelligence; major depressive disorder; knowledge base; explainable artificial intelligence; human digitalisation \| machine learning; suicide prevention; electronic health records; artificial neural networks; risk assessment |
| 3 | 40 | 0.72 | 2020 | breast cancer | artificial intelligence; eating disorders; university students; sleep disturbance; covid-19 pandemic \| breast cancer; aromatase inhibitors; inhibitor-induced arthralgia; postmenopausal women; therapeutic alliance |
| 4 | 50 | 0.757 | 2020 | mobile health | artificial intelligence; systematic review; emotional problems; information ethics; projections \| mental health; mobile health; mobile phone; health application; health intervention |
| 5 | 37 | 0.673 | 2021 | explainable artificial intelligence | artificial intelligence; machine learning; anxiety disorder; facial expression; obsessive-compulsive disorder \| natural language processing; first responders; explainable artificial intelligence; facial expression; risk stratification |
| 6 | 36 | 0.659 | 2022 | depression detection | artificial intelligence; machine learning; feature selection; network pattern recognition; mental disorder \| mental health; deep learning; convolutional neural network; bio-metric signals; long short-term memory |
| 7 | 34 | 0.909 | 2020 | artificial synapse | artificial synapse; resistive switching; conductive filament; analog switching; van der waals heterostructure \| neuromorphic computing; neural network; performance evaluation; behavioral sciences; optical switches |

***Note.*** *Size: the number of articles in each cluster; Silhouette: the average contour value of clustering, it is generally believed that the clustering category with S > 0.5 is reasonable, and S > 0.7 means that the clustering is convincing; LLR: Log-likelihood ratio.*
