# Supplementary material for: Research on the correlation between artificial intelligence and depression: A bibliometric analysis from 2000 to 2024
Source: Medicine (Baltimore). 2026 Sep 18;105(38):e50781. doi: 10.1097/MD.0000000000050781 (PMC13593024; doi:10.1097/MD.0000000000050781)
Supplement: Supplementary file 6 [file medi-105-e50781-s006.docx]

**TABLE S6.** The most trafficked keyword for the top five modules each year.

| **Year** | **2000** | **2001** | **2002** | **2003** | **2004** | **2005** | **2006** | **2007** | **2008** | **2009** |
| --- | --- | --- | --- | --- | --- | --- | --- | --- | --- | --- |
| Total modules | 5 | 5 | 5 | 3 | 4 | 5 | 5 | 8 | 5 | 5 |
| module1 | exposure | pupae | metaanalysis | indians | functional_disability | pertussis | intensification | depression | differential_diagnosis | brief_fatigue_inventory |
| module2 | estrogens | psychiatric_disorders | distortions | order_passeriformes | gender_differences | slow_after-hyperpolarization | intrinsic_signals | follow_up | hypersensitivity | cavum_septum_pellucidum |
| module3 | fat_distribution | housefly_diptera | mood | hemorrhagic_disease | high_pathogenicity | reconstitution | diversity | fecal_incontinence | hydrocortisone | children |
| module4 | evaluative_processes | memory | hydroxide_precipitates | - | depressive_symptoms | sf9_cells | gamblers | deactivation | impact | chemical_speciation |
| module5 | dialysis_encephalopathy | economic_evaluation | frequency_modulation | - | - | r_psychiatric_disorders | jersey_cattle | funnel_chest/diagnosis | ces_d | cell_proliferation |
| **Year** | **2010** | **2011** | **2012** | **2013** | **2014** | **2015** | **2016** | **2017** | **2018** | **2019** |
| Total modules | 7 | 7 | 7 | 11 | 9 | 7 | 6 | 10 | 9 | 12 |
| module1 | dementia | behavior_therapy | breast_cancer | adl_index | alcohol_interactive_medications | brain | alaska_native | anterior_insula | treatment_outcome | perspectives |
| module2 | gambling_subtype | behavioral_intervention | atac_arimidex | antidepressants | visual_disability_variables | adjuvant_treatment | adjuvant_hormonal_therapy | mood_disorders | resilience | psychiatric_disorders |
| module3 | glucocorticoid_replacement | symptoms | balneotherapy | approximate_entropy | item_response_theory | adverse_childhood_experiences | american_indian_adolescents | age-related_macular_degeneration | randomized_controlled_trial | research_domain_criteria |
| module4 | candidate_genes | adult_development | quality_of_life | assisted_living | symptoms | clinical_practice | activation | drug_use | quality_of_life | self_efficacy |
| module5 | cortical_neurons | breast_cancer | associations | aromatase_inhibitor | affective_lability | cognitive_disorders | artificial_intelligence | alcohol_abuse | physical_activity | neurotrophic_factor |
| **Year** | **2020** | **2021** | **2022** | **2023** | **2024** |  |  |  |  |  |
| Total modules | 13 | 15 | 15 | 14 | 17 |  |  |  |  |  |
| module1 | functional_connectivity | acute_coronary_syndrome | suicide_prevention | cognitive_reappraisal | decision_making |  |  |  |  |  |
| module2 | electroconvulsive_therapy | social_support | breast_neoplasms | alaska_native | digital_mental_health |  |  |  |  |  |
| module3 | induced_arthralgia | autobiographical_memory | artificial_neural_networks | psychiatric_disorders | dementia |  |  |  |  |  |
| module4 | historical_trauma | anterior_cingulate_cortex | physical_activity | scoping_review | apps |  |  |  |  |  |
| module5 | neural_network | community | mental_health_care | behavioral_treatment | explainable_artificial_intelligence |  |  |  |  |  |
