# Supplementary material for: Research on the correlation between artificial intelligence and depression: A bibliometric analysis from 2000 to 2024
Source: Medicine (Baltimore). 2026 Sep 18;105(38):e50781. doi: 10.1097/MD.0000000000050781 (PMC13593024; doi:10.1097/MD.0000000000050781)
Supplement: Supplementary file 7 [file medi-105-e50781-s007.docx]

**TABLE S7**. Summary of emerging topics.

| **ClusterID** | **Size** | **Silhouette** | **Average Year** | **Label (LLR)** | **Representative keywords** |
| --- | --- | --- | --- | --- | --- |
| 0 | 118 | 0.889 | 2018 | machine learning | artificial intelligence; electronic health records; decision support; health information technology; family medicine \| machine learning; clinical decision support system; explainable artificial intelligence; neural circuit; artificial neural network |
| 1 | 87 | 0.878 | 2017 | synaptic plasticity | synaptic plasticity; neuromorphic devices; artificial perception systems; ionotronic devices; bionic neural networks \| neuromorphic computing; resistive switching; state variable; neuromorphic applications; artificial neural network |
| 2 | 72 | 0.858 | 2019 | chatbot | artificial intelligence; machine learning; anxiety disorder; stress management; stress relief \| mental health; conversational agent; mobile phone; mobile health; mobile application rating scale |
| 3 | 36 | 0.964 | 2019 | depression detection | artificial intelligence; machine learning; mental disorder; anxiety disorder; patient health questionnaire \| depression detection; video-based facial features; affective computing; bdi-ii estimation; depression diagnosis |
| 7 | 19 | 0.968 | 2020 | deep learning | deep learning; mental health; natural language processing; explainable artificial intelligence; affective computing \| artificial intelligence; machine learning; eating disorders; binge eating; neural network |
| 9 | 16 | 988 | 2020 | neuromorphic computing | neuromorphic computing; ab initio simulation; multilayer memristor; multilevel data-storage memory; electronic synapse \| artificial intelligence; non volatile memory; deep learning; artificial neural networks; multilayer memristor |
| 10 | 16 | 0.971 | 2011 | single | neuromorphic computing; artificial synapse; neural networks; spiking neural network; artificial intelligence \| electronic synapse; pulsed laser deposition; conductive atomic force microscopy; receptor computing; memristive nociceptor |
